# Minimum redundancy maximum relevance feature selection approach for temporal gene expression data

**DOI:** 10.1186/s12859-016-1423-9

**Published:** 2017-01-03

**Authors:** Milos Radovic, Mohamed Ghalwash, Nenad Filipovic, Zoran Obradovic

**Affiliations:** 1 Center for Data Analytics and Biomedical Informatics, College of Science and Technology, Temple University, North 12th Street, Philadelphia, 19122 PA USA; 2 Bioengineering Research and Development Center - BioIRC, Prvoslava Stojanovica 6, Kragujevac, 34000 Serbia; 3 Mathematics Department, Faculty of Science, Ain Shams University, Cairo, 11331 Egypt; 4 Center for Computational Health, IBM T.J. Watson Research Center, Cambridge, MA USA; 5 Faculty of Engineering, University of Kragujevac, Sestre Janjic 6, Kragujevac, 34000 Serbia

**Keywords:** Feature selection, Gene expression, Temporal data

## Abstract

**Background:**

Feature selection, aiming to identify a subset of features among a possibly large set of features that are relevant for predicting a response, is an important preprocessing step in machine learning. In gene expression studies this is not a trivial task for several reasons, including potential temporal character of data. However, most feature selection approaches developed for microarray data cannot handle multivariate temporal data without previous data flattening, which results in loss of temporal information.

We propose a temporal minimum redundancy - maximum relevance (TMRMR) feature selection approach, which is able to handle multivariate temporal data without previous data flattening. In the proposed approach we compute relevance of a gene by averaging F-statistic values calculated across individual time steps, and we compute redundancy between genes by using a dynamical time warping approach.

**Results:**

The proposed method is evaluated on three temporal gene expression datasets from human viral challenge studies. Obtained results show that the proposed method outperforms alternatives widely used in gene expression studies. In particular, the proposed method achieved improvement in accuracy in 34 out of 54 experiments, while the other methods outperformed it in no more than 4 experiments.

**Conclusion:**

We developed a filter-based feature selection method for temporal gene expression data based on maximum relevance and minimum redundancy criteria. The proposed method incorporates temporal information by combining relevance, which is calculated as an average F-statistic value across different time steps, with redundancy, which is calculated by employing dynamical time warping approach. As evident in our experiments, incorporating the temporal information into the feature selection process leads to selection of more discriminative features.

**Electronic supplementary material:**

The online version of this article (doi:10.1186/s12859-016-1423-9) contains supplementary material, which is available to authorized users.

## Background

Feature selection approaches can be roughly categorized into filter-based methods [1], wrapper-based methods [2] and embedded methods [3]. Filter-based methods perform feature selection independently from the learning process. On the other hand, wrapper-based and embedded methods combine feature selection and the learning process in order to select an optimal subset of features. This combined process usually requires the use of nested cross validation procedure which may lead to increased computational cost and possible overfit, especially when a small number of observations is available, which is often the case in gene expression datasets. Therefore, we focus on filter-based feature selection approaches in this paper.

A challenge in gene expression studies is the identification of discriminative genes, which may be later used as predictors (inputs) to classification models. Removing irrelevant features may lead to improved accuracy and increased interpretability of the classification model. However, this task is challenging, especially when data have temporal characteristics. Various feature selection approaches have been developed for microarray data [4–6]. However, most of these methods cannot handle multivariate temporal data without data flattening, which is the process that transforms a temporal data into a single matrix and results in loss of temporal information.

Several feature selection approaches for temporal data have been proposed recently. For instance, [7] proposed a margin-based feature selection approach for temporal data, where the original feature space was transformed into weighted feature space to perform optimization in order to maximize temporal margin in this weighted feature space. However, redundancy among features was not considered. Following the same intuition, in [8, 9] authors proposed an approach, where they project the data to another space to learn new features (factors or principal component). However, the methods are for dimension reduction, rather than feature selection which is our focus in this paper. The Multi-task Lasso method [10, 11] employs group lasso regularization based on the *L*_2,1_-norm penalty for feature selection, thus ensuring all regression models at different time points to share a common set of features. This method removes redundant features by reducing their weights (coefficients) to zero but the approach belongs to the embedded feature selection methods (the search for an optimal subset of features is built into the classifier construction) rather than filter-type methods.

A special group of filter-based feature selection approaches tends to simultaneously select highly predictive but uncorrelated features. An example is the Maximum Relevance Minimum Redundancy (mRMR) algorithm developed for feature selection of microarray data [12]. It tends to select a subset of features having the most correlation with a class (relevance) and the least correlation between themselves (redundancy). In this algorithm, the features are ranked according to the minimal-redundancy-maximal-relevance criteria. Relevance can be calculated by using the F-statistic (for continuous features) or mutual information (for discrete features) and redundancy can be calculated by using Pearson correlation coefficient (for continuous features) or mutual information (for discrete features). In [13] authors proposed the MIFS-ND algorithm, which selects features according to the minimal-redundancy-maximal-relevance criteria by using an optimization algorithm known as Non-dominated Sorting Genetic Algorithm-II [14]. When selecting features, instead of using the calculated values for relevance and redundancy (e.g., F-statistic and Pearson correlation coefficient), authors used domination count and dominated count, which account for the rank in the sorted list of calculated relevance and the rank in the sorted list of calculated redundancy, respectively. In [15], authors proposed an approach, where they select one representative gene from each group/cluster with the objective that the selected genes are jointly discriminative. This approach requires features to be previously clustered based on correlation or domain knowledge (e.g., molecular functions, gene ontology, etc.). By clustering genes this algorithm prevents selection of redundant features. All these algorithms tend to select highly predictive uncorrelated features and require a preprocessing approach to perform temporal data flattening.

In this paper, we propose a temporal minimum redundancy - maximum relevance (TMRMR) feature selection approach, which is able to handle multivariate temporal data without data flattening. We preserve the idea of maximum relevance and minimum redundancy criteria [12] but we change evaluation procedure for relevance and redundancy. In the proposed approach, we compute the relevance of a gene by averaging the F-statistic values calculated across individual time steps, and redundancy between genes by using the dynamical time warping (DTW) approach. The proposed methodology, tested on three temporal gene expression datasets from viral studies, outperforms the alternatives used in this study.

## Methods

### mRMR algorithm and data flattening

**Fig. 1:**
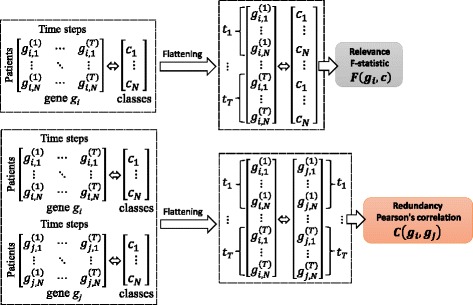
Data flattening commonly used as a preprocessing step to the mRMR

The mRMR is a feature selection approach that tends to select features with a high correlation with the class (output) and a low correlation between themselves. For continuous features, the F-statistic can be used to calculate correlation with the class (relevance) and the Pearson correlation coefficient can be used to calculate correlation between features (redundancy). Thereafter, features are selected one by one by applying a greedy search to maximize the objective function, which is a function of relevance and redundancy. Two commonly used types of the objective function are MID (Mutual Information Difference criterion) and MIQ (Mutual Information Quotient criterion) representing the difference or the quotient of relevance and redundancy, respectively. For temporal data, mRMR feature selection approach requires some preprocessing techniques that flatten temporal data into a single matrix in advance. This may result in a loss of possibly important information among temporal data (such as temporal order information). A common way for data flattening used as a preprocessing step to mRMR is depicted in Fig. 1.

### TMRMR algorithm

**Fig. 2:**
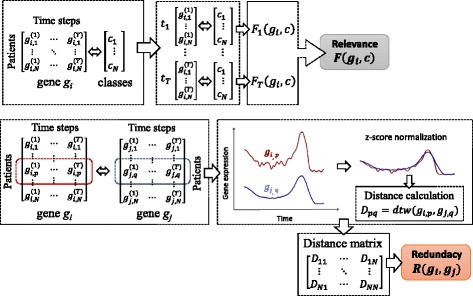
The proposed approach for calculation of relevance and redundancy for temporal data

In this study, we preserve the idea of the mRMR algorithm by maximizing the objective function, which includes relevance and redundancy, but we adapt it to handle multivariate temporal data without flattening (Fig. 2).

Let us denote by $D={{\left \{ {{X}_{i}},{{c}_{i}} \right \}}_{i=1,...,N}}$ the dataset with *N* individuals. ${{X}_{i}}\in {{\mathbf {R} }^{G\times T}}$ represents *G* observed genes measured at *T* time steps for individual *i*. ${{c}_{i}}\in \left \{ 1,...,K \right \}$ represents the class label for individual *i*. Let us also denote by ${{g}_{j}}\in {{\mathbf {R} }^{N\times T}},j=1,...,G$ the *N*×*T* matrix of gene expression data for *j*th gene. We represent relevance of a gene *g*_*j*_ by calculating the F-statistic at each time step and then combining these values by using an appropriate aggregation operator. A number of aggregation operators may be applicable here, such as median, arithmetic mean, geometric mean, maximum or even an approach that combines aggregation operators [16]. However, we aim to choose an operator that is most appropriate for the observed problem, i.e., that is able to capture different gene expression patterns between groups even if differences are present in just a small fraction of the observed time period. Median is more robust to outliers than arithmetic mean which is a nice property, however in some cases it may fail to detect different levels of gene expressions. For instance, some genes may have different expression between groups in just a short time period following infection (e.g., initial two time points with large F-statistic values) and thereafter the differences between groups become insignificant (e.g., the next five time points with small or even neglectable F-statistic values). In such a case, operators like median and geometric mean would fail to detect different gene expression behavior between groups. The maximum F-statistic value may be more appropriate in this case. However, this operator is based on a single F-statistic value (maximal) and it neglects all other values corresponding to other time steps. On the other hand, arithmetic mean, although it will be affected with several small F-statistic values corresponding to the time points where differences in gene expression values between groups do not exist, will have a significant value. In addition, we implemented the Multilayer Aggregation (MLA) method from [16] to combine arithmetic mean, geometric mean and median for aggregation of F-statistic values corresponding to different time steps, however, it did not improve results significantly and it reduced robustness of the proposed feature selection methods. For these reasons, we choose the arithmetic mean operator to aggregate F-statistic values calculated across all time steps into a single value representing the total gene relevance:

1 $$\begin{array}{@{}rcl@{}} F\left(g_{j}^{\left(t \right)},c \right)=\frac{{{\sum\limits_{k=1}^{K}{{{n}_{k}}\left(\bar{g}_{j,(k)}^{\left(t \right)}-\bar{g}_{j}^{\left(t \right)} \right)}}^{2}}/\left(K-1 \right)}{\sum\limits_{k=1}^{K}{\sum\limits_{l=1}^{{n}_{k}}{{{\left(g_{j,l,(k)}^{\left(t \right)}-\bar{g}_{j,(k)}^{\left(t \right)} \right)}^{2}}/\left(N-K \right)}}}. \end{array} $$

2 $$\begin{array}{@{}rcl@{}} F\left({{g}_{j}},c \right)=\frac{1}{T}\sum\limits_{t=1}^{T}{F\left(g_{j}^{\left(t \right)},c \right)} \end{array} $$

where $g_{j}^{\left (t \right)}$ is an *N*-dimensional vector containing gene expression data of a gene *g*_*j*_ at the *t*th time step, *c* is a classification variable with *K* possible classes, *n*_*k*_ is the number of observations belonging to the *k*th class, $\bar {g}_{j}^{\left (t \right)}$ is the average value of $g_{j}^{\left (t \right)}$ in all tissue samples, $\bar {g}_{j,(k)}^{\left (t \right)}$ is the average value of $g_{j}^{\left (t \right)}$ in all tissue samples belonging to the *k*th class, and $g_{j,l,(k)}^{\left (t \right)}$ is the gene expression value of *l*th sample belonging to the *k*th class.

By using Eq. 1, we quantify correlation of a gene *g*_*j*_ with a class at each time step *t*. Thereafter, we calculate the overall relevance of the gene *g*_*j*_ (Eq. 2) by averaging relevance (F-statistic) values calculated for all time steps. Here, it should be noted that relevance calculated in this way differs from relevance calculated on flattened data. For instance, it may happen that for some phenotype 1 expression values for a certain gene have increasing trend (let say from 0 to 1) and for phenotype 2 symmetric decreasing trend (from 1 to 0). In this case, data flattening may lead to low inter-class variance and therefore to low relevance. On the other hand, relevance calculated by using Eqs. (1)-(2) should be able capture the different trends of gene expression data for the two phenotypes.

In the proposed approach for temporal feature selection we calculate redundancy by using DTW, which is an efficient algorithm for measuring similarity between two temporal sequences that may vary in time or speed. DTW uses “elastic” alignment and is able to capture similarity between curves even if they are out of phase in time (in such cases Euclidean and Manhattan distance measures, which align corresponding time points, would fail to detect similarity).

An issue with the mRMR algorithm is the possible selection of irrelevant features, which is possible especially in the first few iterations of the algorithm. For instance, based on the MIQ criterion the second feature may be selected simply because it is totally different from the first one (feature with the highest relevance) although it may be irrelevant. Thereafter, this problem is further propagated since a selected irrelevant feature affects selection of the next ones. In order to solve this issue, we introduced hyperparameter *α*, which controls the number of the top relevant features (according to the average F-statistic value calculated by using Eqs. (1)-(2)) included in the feature selection process. This means that we choose the next non-redundant feature from only the top *α* *G* relevant genes (where *G* is the total number of genes). For each two genes *g*_*i*_ and *g*_*j*_, belonging to the group of the *α* *G* most relevant features, we calculate *N*×*N* distance matrix *D* (Fig. 2) whose elements represent DTW distances between rows in matrices *g*_*i*_ and *g*_*j*_ (e.g., *D*_*pq*_ represents DTW distance between *p*th row in matrix *g*_*i*_ and *q*th row in matrix *g*_*j*_). After computing the distance matrix *D* we calculate redundancy by using one of the following two approaches:

3 $$\begin{array}{@{}rcl@{}} {{R}_{c}}\left({{g}_{i}},{{g}_{j}} \right)=\frac{1}{\frac{1}{{{N}^{2}}}\sum\limits_{p,q}{{{D}_{pq}}}}. \end{array} $$

4 $$\begin{array}{@{}rcl@{}} {{R}_{m}}\left({{g}_{i}},{{g}_{j}} \right)=\frac{1}{\frac{1}{N}\sum\limits_{p=1}^{N}{{{D}_{pp}}}}. \end{array} $$

In Eq. 3 *R*_*c*_ represents redundancy calculated by using DTW distances between every pair of rows in matrices *g*_*i*_ and *g*_*j*_, while in Eq. 4 *R*_*m*_ represents redundancy calculated by using only DTW distances between corresponding rows in matrices *g*_*i*_ and *g*_*j*_.

Although DTW is able to capture similarity between curves that are out of phase in time it may fail to capture similarity between curves fluctuating in a similar manner but with different offsets and amplitudes. For instance, one signal may fluctuate with amplitude between 5 and 10, while another signal may fluctuate in a similar manner but with larger amplitude between 30 and 40. In order to deal with this issue, prior to evaluation of distance matrix *D* for each pair of genes *g*_*i*_ and *g*_*j*_, all gene expression temporal sequences were normalized by the z-score normalization (Fig. 2) which is often used as a preprocessing step to DTW [17–19]:

5 $$\begin{array}{@{}rcl@{}} {{g}_{i,p}}=\frac{\left({{g}_{i,p}}-{{{\bar{g}}}_{i,p}} \right)}{{{\sigma }_{i,p}}}. \end{array} $$

where *g*_*i*,*p*_ is a time series corresponding to *i*th gene and *p*th observation (patient), and ${{\bar {g}}_{i,p}}$ and *σ*_*i*,*p*_ are the average value and standard deviation of this time series. Z-score normalization ’translates’ gene expression time series to fluctuate around the same (zero) offset and removes differences in amplitudes. Thereafter, the gene expression time series differ only in shape which is exactly what we are interested in when calculating redundancy.

After the normalization of gene expression temporal sequences, for each pair of genes *g*_*i*_ and *g*_*j*_ distance matrix *D* is calculated. Each entry of *D* is calculated by using DTW approach:

6 $$\begin{array}{@{}rcl@{}} D_{p,q}= {dtw}\left({{g}_{i,p}},{{g}_{j,q}} \right). \end{array} $$

where *d* *t* *w*() is the function which calculates the DTW distance between temporal sequences *g*_*i*,*p*_ and *g*_*j*,*q*_.

**Fig. 3:**
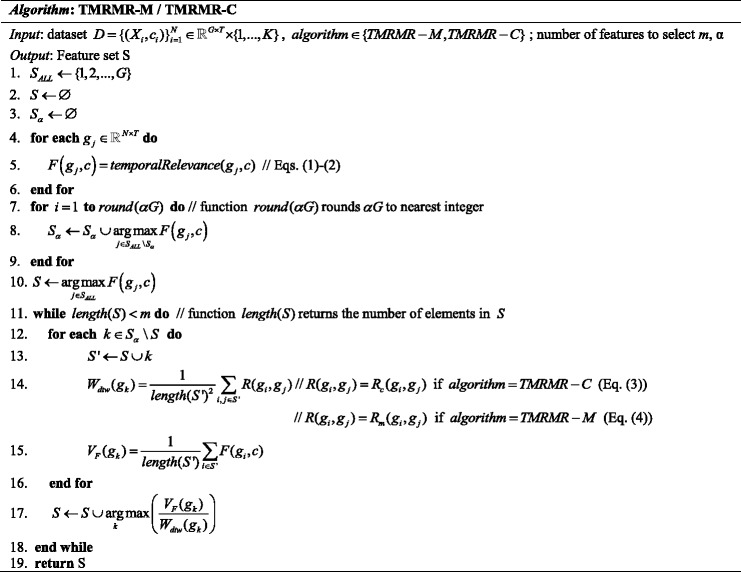
Pseudo code of TMRMR-M and TMRMR-C feature selection algorithms

As in mRMR [12], the proposed algorithm starts by selecting one feature (gene) having the largest relevance calculated by using Eq. 2. Thereafter, algorithm performs greedy search and adds one feature in each iteration according to the MIQ criterion:

7 $$\begin{array}{*{20}l} \underset{{{g}_{k}}}{\mathop{\max }}\,\left(\frac{{{V}_{F}}}{{{W}_{dtw}}} \right),{{V}_{F}}&=\frac{1}{\left| S \right|}\sum\limits_{i\in S}{F\left({{g}_{i}},c \right)},{{W}_{dtw}}\\ &=\frac{1}{{{\left| S \right|}^{2}}}\sum\limits_{i,j\in S}{R\left({{g}_{i}},{{g}_{j}} \right)}. \end{array} $$

where *S* is a subset of already selected genes extended with gene *g*_*k*_ and $\left | S \right |$ is the number of features in *S*, *F* is the average F-statistic value across different time steps (Eq. 2), and *R* is either *R*_*c*_ (Eq. 3) or *R*_*m*_ (Eq. 4). Depending on the choice of the redundancy measure (*R*_*c*_ or *R*_*m*_), in this paper we propose two versions of the TMRMR algorithm: (1) TMRMR-C, using *R*_*c*_ as a measure of redundancy and (2) TMRMR-M, using *R*_*m*_ as a measure of redundancy. Figure 3 shows the pseudo-code of the proposed TMRMR-C and TMRMR-M algorithms.

Solution to the optimization problem given in Eq. 7 requires *O*(*α* *G* *m*) computational complexity, where *m* is the number of genes selected. Taking into account that the computational complexity of the DTW algorithm is *O*(*T* ^2^) then the total time complexities of the TMRMR-C and TMRMR-M algorithms are *O*(*α* *G* *m* *T* ^2^ *N* ^2^) and *O*(*α* *G* *m* *T* ^2^ *N*), respectively. Both proposed algorithms require more computational complexity than the original mRMR algorithm whose computational complexity is *O*(*G* *m* *T* *N*) for the temporal gene expression dataset. However, in cases where it is necessary to reduce execution time of the proposed algorithms (e.g. datasets with large number of time points *T*), their computational complexity may be reduced through parameter *α*. In addition, we can further speed up the proposed algorithms by utilizing an approximate DTW that has a linear time complexity [20], however, it is out of the current manuscript’s scope.

### Implementation

Both, the TMRMR-C and TMRMR-M algorithms are implemented by using MATLAB software. DTW is implemented by using dynamic time warping package [21]. Our software takes as input a set of temporally aligned gene expression data and provides the ranked list of the top genes as the output. The number of genes to be selected is specified by a user. Source code is freely available at: https://github.com/radovicmiloskg/TMRMR.git.

## Results and discussion

### Dataset description

In this study, we evaluated the proposed feature selected approach by comparing it with alternatives on three independent gene expression datasets from human viral challenge studies [22]. These datasets contain gene expression data for 17, 20 and 19 human volunteers, who were infected with H3N2 influenza, rhinovirus (HRV) and respiratory syncytial virus (RSV), respectively. A summary of the datasets is given in Table 1.

**Table 1:** Description of gene expression datasets

Dataset	# Genes	# Samples (symptomatic/	No of time points
		asymptomatic)	
H3N2	12023	17 (9/8)	16
HRV	12023	20 (10/10)	14
RSV	12023	19 (9/10)	21

In each dataset, subjects were classified based on severity of reaction to infection into “symptomatic” and “asymptomatic” groups. In particular, symptoms were recorded twice daily and classified based on modified Jackson Score [23]. Patients with a modified Jackson score larger than or equal to 6 over the quarantine period were denoted as “symptomatic”. Gene expression measurements were collected temporally, starting at baseline (24 hours prior to inoculation with virus) and thereafter at a certain time points following experimental procedure which is described in detail in [22], making a total of 16, 14 and 21 time-point measurements for H3N2, HRV and RSV datasets, respectively.

### Comparison methods

We compared the proposed TMRMR-C and TMRMR-M methods with four popular state-of-the-art feature selection approaches, widely used for extraction of the most informative features from gene expression data:

1. **mRMR**: This algorithm tends to select a subset of features having the most correlation with the class (output) and the least correlation between themselves [12]. It ranks features according to the minimal-redundancy-maximal-relevance criterion which is based on mutual information.
2. **F-statistic**: ANOVA is one of the most widely applied techniques in microarray data analysis [24]. This approach selects features simply according to the F-statistic value (which is the statistic for ANOVA). It prefers to select features having small intra-class variances and large inter-class variance.
3. **ReliefF**: One of the most successful and most widely used feature selection approaches which is based on the idea that a good feature should have similar values in observations belonging to the same class and different values in observations belonging to different classes [25]. It choses instances randomly, finds their nearest neighbors from the same and the opposite class(es), and weights features according to their distances (more weight is given to features that discriminate the instances from neighbors of different class(es) and do not discriminate the instances from neighbors of the same class).
4. **Multi-task Lasso (MT-LASSO)**: This method represents one of the state-of-the-art methods for temporal feature selection [10, 11]. It employs the group lasso regularization based on the *L*_2,1_-norm penalty for feature selection, thus ensuring that all regression models at different time points (tasks) share a common set of features. The method is implemented by using the MALSAR software package [26].

### Performance evaluation procedure

We evaluated the feature selection approaches by calculating the classification accuracy of the three classifiers:

1. **K-nearest neighbors (KNN)**: Instance-based lazy learning algorithm which predicts the class of a testing observation that is dominant among the K most similar examples (nearest neighbors) in the problem space.
2. **Naive Bayes classifier (NB)**: A probabilistic classifier based on applying Bayes’ theorem, which is often used for classification of gene expression data [12, 27].
3. **Support vector machine (SVM)**: A discriminative classifier, which uses a kernel trick to transform the input data space in order to create a separating hyperplane. In this study, we used linear SVM because previous studies have proved its effectiveness in gene expression classification problems [28].

For evaluation of the three classifiers, the 5-fold cross validation procedure was used, where, in each iteration, observations belonging to the left-out fold were used for testing purposes (test set), while the remaining observations were used for feature selection followed by classifier training (training set). In each iteration of the cross validation procedure we optimized parameters of the classifiers by applying nested 5-fold cross validation procedure on the training set. In this way optimal values of parameters $C\in \left \{ {{10}^{-3}},{{10}^{-2}},...,{{10}^{3}} \right \}$ for SVM and $K\in \left \{ 1,3,5,7 \right \}$ for KNN were selected. Here it should be noted that the test data were never used for feature selection and classifiers training (including optimization of classifiers parameters - nested 5-fold cross validation procedure). In addition, the optimal value of the hyperparameter *α* can be estimated in a nested cross-validation procedure. However, due to the fact that datasets used in this study contain a huge number of features (12023) measured in a large number of time points (14-21 depending on a dataset), it was too time consuming to use the nested cross-validation to select the value of *α*. Thus, we simply fixed the *α* parameter to 0.3 in all experiments ensuring that all selected genes come from the pool of the top 30% relevant genes (3610 genes).

All three gene expression datasets used in this study are balanced, and therefore classification accuracy may serve as a good metric for comparison of TMRMR-C and TMRMR-M with other baseline feature selection methods. Prior to feature selection and evaluation, missing values in all three datasets were imputed by linear interpolation. In addition, gene expression values for each gene were normalized to the range [ 0,1] by using min-max normalization. All methods were implemented by using MATLAB software.

### Classification accuracy on gene expression data

The proposed TMRMR-C and TMRMR-M feature selection approaches were compared with four baseline feature selection algorithms according to the evaluation procedure described in the previous section. By using the 5-fold cross validation procedure, the accuracy of KNN, NB and SVM classifiers was calculated for the top *m*={1,10,20,30,40,50} genes.

Table 2 shows the results for the three datasets H3N2, HRV and RSV, respectively. It clearly shows that both TMRMR-C and TMRMR-M methods outperformed alternatives in most cases. More precisely, both TMRMR-C and TMRMR-M algorithms achieved improvement in 34 out of 54 cases when compared to alternatives (with 12 tie results). When comparing the two proposed approaches, TMRMR-C outperformed TMRMR-M in most cases (16-5 in favor of TMRMR-C and 33 tie results). These results reveal that redundancy calculated by using DTW distances between every pair of time series from gene expression matrices (*R*_*c*_) significantly contributes to prediction accuracy. In addition, we calculated the average accuracy of the three classifiers over all datasets (last row in Table 2). These values show that, on average, both proposed methods outperformed alternatives in all cases (for all classifiers and all *m* values). This indicates that the proposed methods have selected the most discriminative features.

**Table 2:** Evaluation of feature selection methods on H3N2, HRV and RSV datasets using the top m genes (values represent classification accuracy)

	Feature	KNN							NB						SVM							
	selection	Number of features							Number of features						Number of features							
	method	1	10	20	30	40	50		1	10	20	30	40	50		1	10	20	30	40	50	
H3N2	mRMR	58.8	76.5	82.4	88.2	88.2	88.2		64.7	76.5	76.5	70.6	70.6	76.5		58.8	70.6	64.7	70.6	76.5	88.2	
	F-statistic	58.8	82.4	88.2	88.2	88.2	94.1		64.7	82.4	88.2	94.1	94.1	94.1		58.8	88.2	88.2	88.2	88.2	100	
	ReliefF	64.7	47.1	70.6	76.5	82.4	82.4		70.6	52.9	82.4	88.2	88.2	94.1		52.9	70.6	94.1	100	94.1	94.1	
	MT-LASSO	52.9	70.6	76.5	94.1	88.2	100		64.7	70.6	64.7	76.5	82.4	76.5		58.8	82.4	70.6	94.1	100	100	
	TMRMR-C	100	100	100	100	100	100		94.1	100	100	100	100	100		88.2	100	94.1	94.1	94.1	100	
	TMRMR-M	100	100	100	100	100	100		94.1	94.1	100	100	100	100		94.1	100	94.1	94.1	100	100	
HRV	mRMR	40.0	40.0	50.0	60.0	55.0	60.0		35.0	40.0	65.0	60.0	75.0	75.0		35.0	40.0	70.0	65.0	60.0	65.0	
	F-statistic	40.0	55.0	85.0	75.0	75.0	75.0		35.0	75.0	70.0	70.0	80.0	80.0		30.0	60.0	70.0	85.0	75.0	80.0	
	ReliefF	45.0	55.0	55.0	55.0	60.0	60.0		50.0	50.0	40.0	50.0	50.0	60.0		55.0	50.0	45.0	50.0	60.0	60.0	
	MT-LASSO	40.0	50.0	50.0	65.0	60.0	60.0		40.0	55.0	60.0	70.0	75.0	75.0		40.0	55.0	50.0	60.0	70.0	75.0	
	TMRMR-C	55.0	80.0	80.0	75.0	85.0	75.0		50.0	75.0	85.0	90.0	85.0	80.0		50.0	75.0	85.0	85.0	75.0	75.0	
	TMRMR-M	55.0	60.0	75.0	75.0	80.0	75.0		50.0	75.0	85.0	80.0	80.0	80.0		50.0	70.0	80.0	75.0	80.0	75.0	
RSV	mRMR	84.2	68.4	68.4	63.2	63.2	68.4		79.0	68.4	68.4	63.2	57.9	68.4		84.2	68.4	57.9	57.9	57.9	57.9	
	F-statistic	79.0	63.2	68.4	63.2	63.2	63.2		79.0	68.4	79.0	73.7	57.9	68.4		84.2	73.7	79.0	68.4	68.4	63.2	
	ReliefF	73.7	47.4	36.8	31.6	36.8	42.1		68.4	68.4	79.0	52.6	47.4	47.4		68.4	68.4	52.6	47.4	47.4	42.1	
	MT-LASSO	79.0	57.9	52.6	47.4	57.9	57.9		79.0	89.5	73.7	63.2	57.9	52.6		79.0	73.7	57.9	52.6	52.6	57.9	
	TMRMR-C	79.0	84.2	73.7	84.2	84.2	84.2		79.0	84.2	84.2	84.2	84.2	84.2		79.0	84.2	79.0	89.5	84.2	84.2	
	TMRMR-M	79.0	84.2	84.2	73.7	73.7	73.7		79.0	84.2	84.2	84.2	84.2	84.2		79.0	73.7	84.2	73.7	79.0	79.0	
Average	mRMR	61.0	61.6	66.9	70.5	68.8	72.2		59.6	61.6	70.0	64.6	67.8	73.3		59.3	59.7	64.2	64.5	64.8	70.4	
	F-statistic	59.3	66.8	80.6	75.5	75.5	77.4		59.6	75.3	79.1	79.3	77.3	80.8		57.7	74.0	79.1	80.6	77.2	81.1	
	ReliefF	61.1	49.8	54.1	54.4	59.7	61.5		63.0	57.1	67.1	63.6	61.9	67.2		58.8	63.0	63.9	65.8	67.2	65.4	
	MT-LASSO	57.3	59.5	59.7	68.8	68.7	72.6		61.2	71.7	66.1	69.9	71.7	68.0		59.3	70.3	59.5	68.9	74.2	77.6	
	TMRMR-C	**78.0**	**88.1**	84.6	**86.4**	**89.7**	**86.4**		**74.4**	**86.4**	**89.7**	**91.4**	**89.7**	**88.1**		72.4	**86.4**	86.0	**89.5**	84.4	**86.4**	
	TMRMR-M	**78.0**	81.4	**86.4**	82.9	84.6	82.9		**74.4**	84.4	**89.7**	88.1	88.1	**88.1**		**74.4**	81.2	**86.1**	80.9	**86.3**	84.7	

Bold represents the best average accuracy

For each *m* value, we tested whether the proposed TMRMR-C approach (which outperformed the TMRMR-M) statistically significantly outperforms other methods. For this purpose, we applied Welch’s t-test on the results given in Table 2 and found that the accuracy of the proposed TMRMR-C method is statistically more significant than other four baseline methods in 17 out of 24 cases (*α*=0.05).

**Fig. 4:**
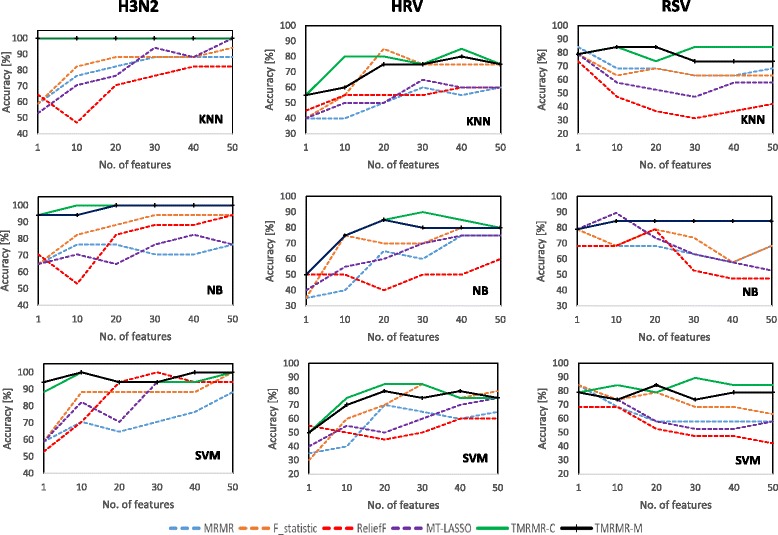
Classification accuracy obtained by using 5-fold cross validation procedure on the three gene expression datasets: H3N2 (*left*), HRV (*middle*) and RSV (*right*). Results are given for the three classifiers: KNN (*top*), NB (*middle*) and SVM (*down*)

Results given in Table 2 are depicted in Fig. 4. In this figure the accuracy is plotted as a function of *m* for all classifiers and for all datasets. This figure clearly shows that in most cases both, TMRMR-C and TMRMR-M approaches, outperform baseline methods for most values of *m*. This figure also shows that, among the four tested baseline feature selection approaches, F-statistic outperformed the others in most cases including mRMR. Since mRMR uses F-statistic as a measure of relevance we can conclude that minimum redundancy condition, calculated as a Pearson correlation coefficient, hurts its performance. On the other hand, the proposed TMRMR-C and TMRMR-M methods achieved highest accuracy by combining relevance, calculated as an average F-statistic value across different time steps, with redundancy, calculated by employing DTW and thus succeeded to capture some additional information hidden in temporal characteristics of the data.

**Fig. 5:**
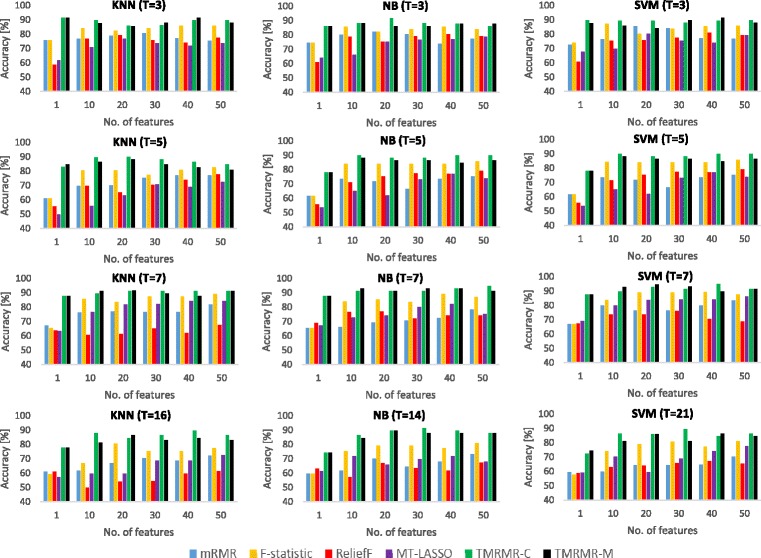
Average classification accuracy over all datasets obtained in a 5-fold cross validation procedure. Results are given for a different number of time points used for both feature selection and classifiers training: *T*=3, *T*=5, *T*=7 and *T*=*T*_*all*_ where *T*_*all*_∈{16,14,21}

The accuracy of the DTW algorithm may degrade considerably when operating on expression profiles with not enough data points which is often the case in gene expression datasets. This may limit the applicability of the proposed TMRMR-C and TMRMR-M algorithms in such cases and, for this purpose, we performed analysis on how reducing the number of time points affects performance of the proposed methods comparing to baseline approaches. We repeated the same evaluation procedure but with reduced number of time points *T*=3, *T*=5 and *T*=7 for all three datasets. We select the following time points for evaluation purposes: initial time point, end time point and equally distant time points between them (e.g. *t*_1_, *t*_*T*/2_, *t*_*T*_). Due to the space limitation, in Table 3 and Fig. 5 we show only results averaged over all datasets. The obtained results show that the reduction of time points did not affect the performance of the TMRMR-C algorithm, which outperformed all alternatives in all cases (for all classifiers and for all *T* and *m* values). On the other hand, the TMRMR-M algorithm showed improvement in all but 3 cases from which 2 occurred when the number of time points was set to 3 (*T*=3) and the remaining one occurred when the number of time points was set to 5 (*T*=5). This confirms the fact that a limited number of time points negatively affects the DTW approach and consequently the TMRMR-M algorithm, nevertheless, the proposed method showed improvement in most cases when comparing to baseline approaches. This leads to the conclusion that in cases with a limited number of time points the TMRMR-C approach, which is computationally more expensive, might be more appropriate than the TMRMR-M approach.

**Table 3:** Evaluation of feature selection methods on H3N2, HRV and RSV datasets using the top m genes and reduced number of time points (T=3, T=5 and T=7). Values represent average accuracy on the three datasets obtained by using five-fold cross validation procedure

	Feature	KNN							NB							SVM						
	selection	Number of features							Number of features							Number of features						
	method	1	10	20	30	40	50		1	10	20	30	40	50		1	10	20	30	40	50	
T=3	mRMR	75.8	76.6	79.0	80.8	77.3	75.5		74.4	80.2	82.3	80.6	73.8	77.1		72.5	76.5	85.6	84.2	77.0	77.0	
	F-statistic	75.8	84.1	82.3	84.2	85.9	85.9		74.4	85.8	82.1	84.1	85.7	83.8		74.2	87.5	80.3	83.7	85.5	86.0	
	ReliefF	58.6	76.7	79.2	75.7	74.0	77.6		60.8	78.5	75.2	78.9	80.6	79.1		60.8	75.3	75.8	77.4	81.1	79.1	
	MT-LASSO	61.6	71.0	76.8	73.7	72.0	73.8		64.1	66.0	75.2	76.5	76.7	78.6		67.8	69.9	80.5	75.5	74.0	79.2	
	TMRMR-C	**91.3**	**89.7**	**86.0**	86.3	89.7	**89.7**		**86.1**	88.1	**91.5**	**88.0**	**87.8**	86.1		**89.7**	**89.4**	**89.4**	88.0	89.4	**89.7**	
	TMRMR-M	**91.3**	87.8	85.7	**88.0**	**91.3**	88.0		**86.1**	**88.2**	86.0	86.0	**87.8**	**87.8**		87.7	85.8	84.1	**89.7**	**91.4**	88.1	
T=5	mRMR	61.1	69.8	69.9	75.4	76.9	77.0		61.5	73.5	71.8	66.6	73.7	75.4		64.9	73.3	68.1	73.8	79.0	77.2	
	F-statistic	61.1	80.6	80.6	77.2	80.8	82.6		61.5	84.2	84.1	84.1	84.1	85.7		64.9	84.1	80.6	79.0	78.9	84.3	
	ReliefF	55.3	69.7	65.0	70.6	74.0	77.7		55.6	71.2	75.3	77.2	77.1	79.1		57.0	72.0	72.1	70.4	72.0	70.1	
	MT-LASSO	49.8	55.8	63.1	70.7	69.0	72.4		53.8	65.0	62.1	73.3	76.9	73.8		51.5	68.0	70.4	77.7	74.4	76.1	
	TMRMR-C	83.0	**89.7**	**89.7**	**88.0**	**86.3**	**84.6**		**77.9**	**89.7**	**88.1**	**88.1**	**89.7**	**89.7**		81.3	**91.3**	**88.0**	**86.3**	**87.6**	**86.4**	
	TMRMR-M	**84.7**	86.3	88.0	84.7	82.7	81.0		**77.9**	88.1	86.4	86.4	84.7	86.4		**83.0**	88.0	**88.0**	82.8	86.1	84.4	
T=7	mRMR	67.3	76.3	77.0	76.7	76.6	81.8		65.3	66.1	69.1	70.7	72.3	78.4		67.1	80.0	76.5	76.5	80.1	83.6	
	F-statistic	65.5	85.6	83.6	87.3	87.5	89.3		65.3	83.8	85.4	83.5	89.1	87.1		67.1	84.0	89.1	89.1	89.3	87.6	
	ReliefF	63.7	60.5	61.3	65.2	61.8	67.4		69.0	76.7	76.9	72.3	73.9	74.2		67.3	73.8	73.7	76.0	70.7	68.8	
	MT-LASSO	63.3	76.5	81.9	82.3	84.2	84.2		67.3	72.7	74.1	80.2	82.1	75.1		69.0	79.8	83.7	84.1	84.1	86.1	
	TMRMR-C	**87.7**	89.6	91.3	**91.3**	**91.3**	**91.3**		**87.7**	91.3	**91.3**	91.3	93.0	**94.7**		87.7	89.7	93.0	91.3	**94.8**	**91.3**	
	TMRMR-M	**87.7**	**91.3**	**91.4**	89.6	87.8	**91.3**		**87.7**	**93.1**	**91.3**	**93.0**	**93.1**	91.3		**87.8**	**93.0**	**94.7**	**93.1**	89.6	**91.3**	

Bold represents the best average accuracy

### Gene ontology over-representation analysis

We have performed gene ontology over-representation analysis to find gene ontology (GO) terms that are over-represented within the subset of selected genes. For this purpose we used annotations for the top 50 genes selected by the TMRMR-C algorithm from each of the three datasets used in this study (the full list of selected genes, together with error bars for the two groups, symptomatic and asymptomatic, is given in Additional file 1). Selected genes from each dataset were independently submitted to the PANTHER (protein annotation through evolutionary relationship) classification system (http://www.pantherdb.org/) which extracted significantly over-represented biological processes. For each of the three datasets, the top 5 GO terms are reported in Table 4. The last column in the table is *p*-value corrected based on the Bonferroni procedure.

**Table 4:** Top 5 GO terms over-represented in the top 50 genes selected by the TMRMR-C algorithm from H3N2, HRV and RSV datasets

Dataset	GO ID	GO biological process	*P*-value
H3N2	GO:0060337	Type I interferon signaling pathway	6.17E–23
	GO:0071357	Cellular response to type I interferon	6.17E–23
	GO:0034340	Response to type I interferon	1.55E–22
	GO:0051607	Defense response to virus	2.52E–22
	GO:0009615	Response to virus	6.85E-21
HRV	GO:0060337	Type I interferon signaling pathway	2.51E–18
	GO:0071357	Cellular response to type I interferon	2.51E–18
	GO:0034340	Response to type I interferon	5.56E–18
	GO:0009615	Response to virus	2.08E–15
	GO:0051607	Defense response to virus	1.07E–14
RSV	GO:0070269	Pyroptosis	1.46E–03
	GO:0002376	Immune system process	1.93E–03
	GO:0006955	Immune response	1.95E–03
	GO:0045087	Innate immune response	3.68E–03
	GO:0006952	Defense response	6.96E–03

We can see from Table 4 that most of GO terms that are over-represented in all datasets are related to immune response to viral infection. This is consistent with the fact that the three gene expression datasets originate from human viral challenge studies where human volunteers were infected with H3N2 influenza, rhinovirus (HRV) and respiratory syncytial virus (RSV), respectively.

### Robustness

**Fig. 6:**
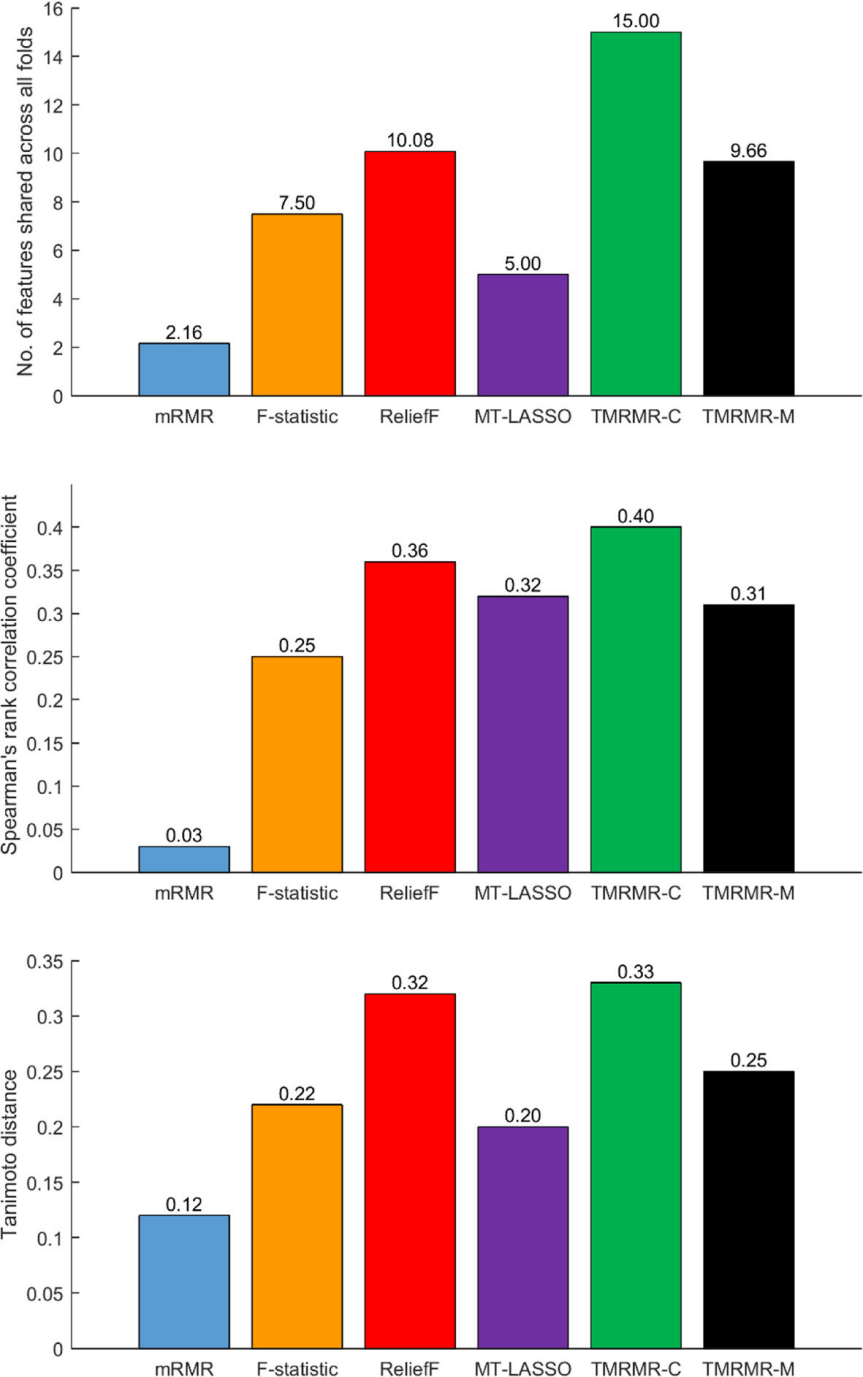
Robustness analysis. The average values of Spearman’s rank correlation coefficient (*ρ*), Tanimoto distance (*T*_*dist*_) and number of features shared across all folds (*N*_*shared*_) for all experiments (all datasets and all tested number of time points)

In order to compare robustness of the proposed TMRMR-C and TMRMR-M feature selection methods with other baseline approaches used in this study, we calculated the Spearman’s rank correlation coefficient (*ρ*), Tanimoto distance (*T*_*dist*_) and number of features shared across all folds of the 5-fold cross validation procedure (*N*_*shared*_) for the top 50 selected features (*m*=50). For each method, Fig. 6 shows the average value of each stability measure across all datasets (H3N2, HRV and RSV) and across all tested numbers of time points (*T*=3, *T*=5, *T*=7 and *T*=*T*_*all*_, where *T*_*all*_∈{16,14,21}). This figure clearly shows that, on average, the TMRMR-C feature selection method is the most stable one according to each of the three measures (*N*_*shared*_=15, *ρ*=0.40 and *T*_*dist*_=0.33). The second most stable method is ReliefF (*N*_*shared*_=10.08, *ρ*=0.36 and *T*_*dist*_=0.32) which appears to be more stable than the TMRMR-M algorithm (*N*_*shared*_=9.66, *ρ*=0.31 and *T*_*dist*_=0.25), while the least stable method is mRMR (*N*_*shared*_=2.16, *ρ*=0.03 and *T*_*dist*_=0.12). Since both the mRMR and the TMRMR-C algorithms are based on maximum relevance and minimum redundancy criteria, we can conclude that combining relevance, calculated as an average F-statistic value across different time steps, with redundancy, calculated by employing DTW significantly improves robustness for temporal data.

## Conclusion

We presented filter-based feature selection methods for temporal gene expression data. The proposed methods utilize the maximum relevance and minimum redundancy criteria which were originally introduced by the mRMR algorithm. In order to handle multivariate temporal data without previous data flattening we modified the evaluation procedure for relevance and redundancy. Concretely, in the proposed methods we calculate the relevance of a gene by averaging F-statistic values calculated across individual time steps and redundancy between genes by using dynamical time warping. The proposed methods have been tested on three temporal gene expression datasets from viral studies. We showed that TMRMR-C and TMRMR-M proposed methods outperformed alternatives in most cases. In addition, we evaluated the proposed approaches on a reduced number of time points and showed that they achieved improvement in most cases when compared to alternatives. In the future, we will focus on optimization of minimum-redundancy-maximum-relevance criteria and investigate applicability of various optimization algorithms, other than greedy search used in this study.

## Additional file

Additional file 1 Supplementary materials. The supplementary PDF file contains relevant information omitted from the main manuscript such as: (1) the ranked list of the top 50 genes selected by the TMRMR-C approach for H3N2, HRV and RSV datasets, respectively and (2) error bars for the two groups, symptomatic and asymptomatic, for the top genes selected from the three datasets. (DOCX 240 kb)

## Abbreviations

DTW: Dynamical time warping
GO: Gene ontology
H3N2: H3N2 influenza virus
HRV: Rhinovirus
KNN: K-nearest neighbors
MID: Mutual Information difference criterion
MIQ: Mutual Information quotient criterion
mRMR: Maximum relevance minimum redundancy algorithm
MT-LASSO: multi-task Lasso
NB: Naive bayes classifier
PANTHER: Protein annotation through evolutionary relationship
RSV: Respiratory syncytial virus
SVM: Support vector machine
TMRMR: Temporal minimum redundancy maximum relevance feature selection method
