# Supplementary material for: Minimum redundancy maximum relevance feature selection approach for temporal gene expression data
Source: BMC Bioinformatics. 2017 Jan 3;18:9. doi: 10.1186/s12859-016-1423-9 (PMC5209828; doi:10.1186/s12859-016-1423-9)
Supplement: Supplementary file 1 — Supplementary materials. The supplementary PDF file contains relevant information omitted from the main manuscript such as: (1) the ranked list of the top 50 genes selected by the TMRMR-C approach for H3N2, HRV and RSV datasets, respectively and (2) error bars for the two groups, symptomatic and asymptomatic, for the top genes selected from the three datasets. (DOCX 240 kb) [file 12859_2016_1423_MOESM1_ESM.docx]

**Supplementary Material for Minimum redundancy maximum relevance feature selection approach for temporal gene expression data**

Since the TMRMR-C method showed improvement in accuracy and higher stability than the TMRMR-M method, we focus on genes selected by this approach. Here we provide the ranked list of the top 50 genes selected by the TMRMR-C approach for H3N2, HRV and RSV datasets, respectively (Table S1). For each dataset, these genes were independently submitted to the PANTHER (protein annotation through evolutionary relationship) classification system (<http://www.pantherdb.org/>) which extracted significantly over-represented biological processes. These results are given in the main document.

In addition, Figures S1-S3 show error bars for the two groups, symptomatic and asymptomatic, for the top nine genes selected from H3N2, HRV and RSV datasets, respectively.

Table S1: Top 50 genes selected by the TMRMR-C algorithms for H3N2, HRV and RSV datasets, respectively

| **Dataset** | **Selected genes** |
| --- | --- |
| H3N2 | LY6E, LOC130074, ISG15, OAS1, SERPING1, IFI44L, ENOSF1, RTP4, IFI44, SCO2, IFITM3, CERK, RSAD2, SIGLEC1, EIF3EIP, IFIT3, MT2A, LOC26010, CBX7, IFIT1, CD1C, PLSCR1, OAS2, APOL6, GRAMD1C, XAF1, OAS3, STAT1, CNP, IL16, HERC5, GBP1, QARS, IFITM1, IRF7, CIRBP, LAP3, UBE2L6, EIF2AK2, RPLP1, RNF44, MX1, IFI6, ZCCHC2, EEF2, OASL, SAMD4A, RPS16, LILRB2, C13orf18 |
| HRV | ASF1B, RSAD2, GSTK1, OAS1, TPST1, XAF1, CNDP2, GPM6A, OAS2, ARHGAP19, IFI44L, HLA-DRB1, LOC26010, IFI44, PISD, PPP4R1, MX1, EMR3, LY6E, BTG2, DTX4, IFIT3, UPB1, IFI6, CHI3L1, HERC5, LDLR, BST2, OGFRL1, RGL2, PSMB9, PPP2R5A, OAS3, IFI35, PKP4, GBP1, MMP9, CABC1, SAMHD1, IRF9, SRPK2, CNP, EPB41L3, IFI16, SIGLEC1, RPGRIP1, GM2A, NECAB2, OASL, MME |
| RSV | CD177, C1QA, PASK, C1QB, C11orf75, FOXO1, LOC391020, PLSCR1, FADS3, SQRDL, STAT2, SERPINB1, ZNF266, AIM2, CENTA2, TXNDC3, STX11, SERTAD2, BLVRA, BST2, CASP5, TYMP, ALDH9A1, RTP4, TLR5, VPREB3, IFI6, APOBEC3A, CEACAM1, C11orf80, UBE2L6, PARP12, SPIN1, SERPING1, C13orf18, IFI35, PHF1, STK3, CD22, CCR1, CD200, CASP1, LOC26010, TULP3, FLVCR2, VAMP5, SPOP, APOL2, NPC2, DTX4 |


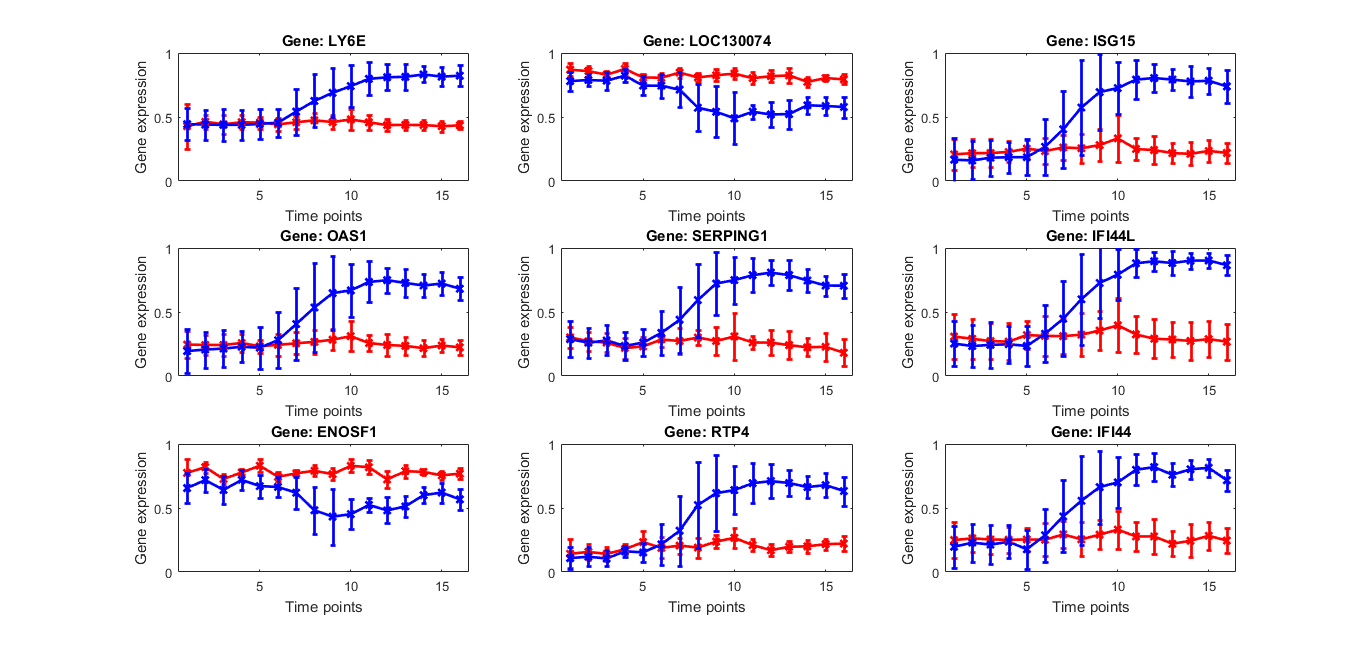
Figure S1: Top nine genes selected by the TMRMR-C algorithm from the H3N2 dataset. Solid lines and error bars represent the mean and the standard deviation of the gene expression over all subjects in (1) asymptomatic (blue) and (2) symptomatic (red) group.


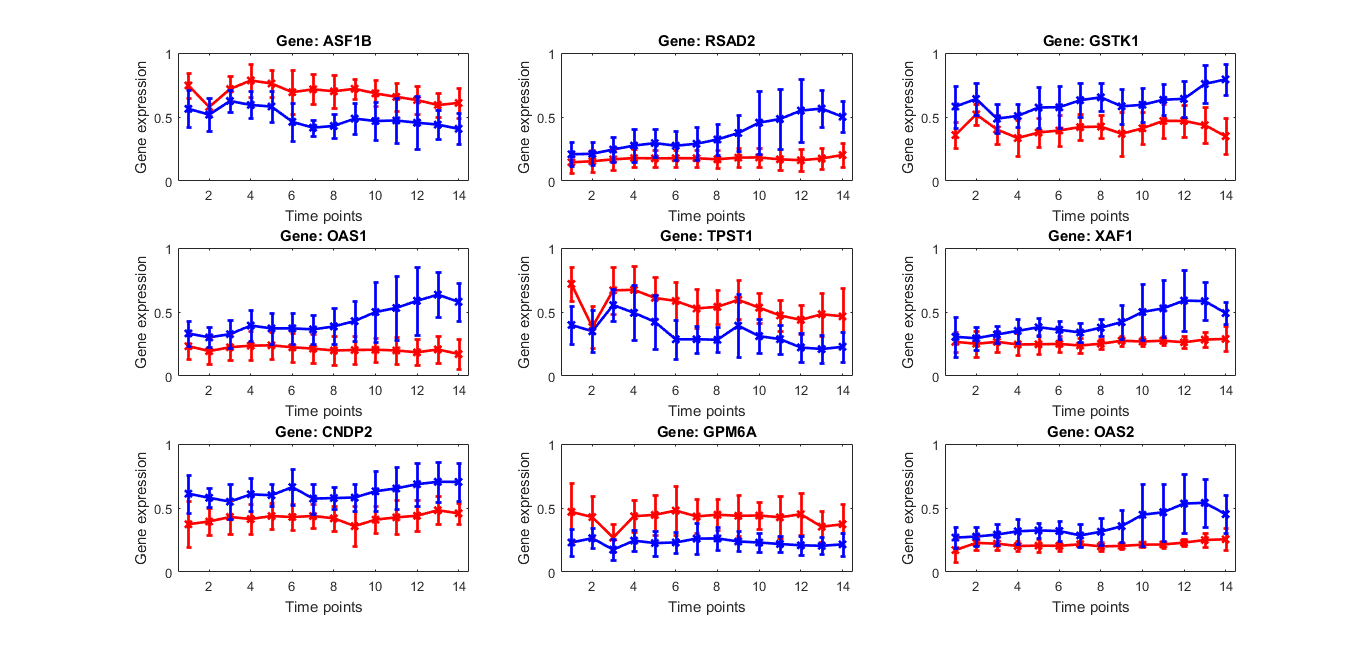
Figure S2: Top nine genes selected by the TMRMR-C algorithm from the HRV dataset. Solid lines and error bars represent the mean and the standard deviation of the gene expression over all subjects in (1) asymptomatic (blue) and (2) symptomatic (red) group.


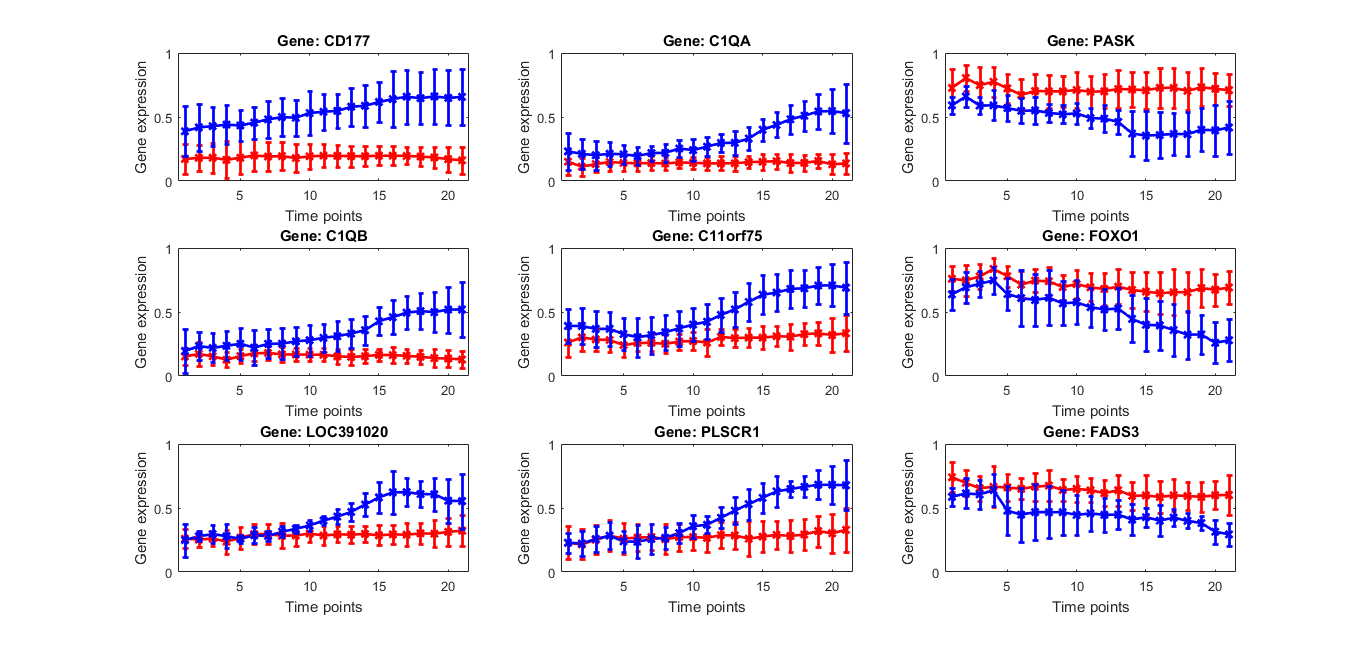
Figure S3: Top nine genes selected by the TMRMR-C algorithm from the RSV dataset. Solid lines and error bars represent the mean and the standard deviation of the gene expression over all subjects in (1) asymptomatic (blue) and (2) symptomatic (red) group.
